# Supplementary material for: Detection of lymphoproliferative disease virus in Iowa Wild Turkeys (Meleagris gallopavo): Comparison of two sections of the proviral genome
Source: PLoS One. 2024 Feb 12;19(2):e0296856. doi: 10.1371/journal.pone.0296856 (PMC10861079; doi:10.1371/journal.pone.0296856)

Supporting Information for “Detection of lymphoproliferative disease virus in Iowa Wild Turkeys (*Meleagris gallopavo*): Comparison of two sections of the proviral genome” by Smith & Blanchong

**Gel 1.** Representative agarose gel image of amplification of a 413-nucleotide sequence of lymphoproliferative disease virus proviral DNA in Iowa Wild Turkeys covering parts of the p31 and capsid regions of the *gag* gene.


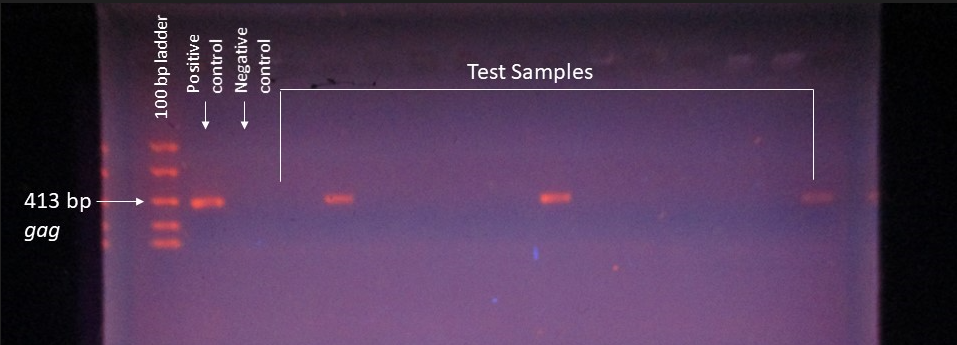


**Gel 2.** Representative agarose gel image of amplification of a 335-nucleotide sequence of lymphoproliferative disease virus proviral DNA in Iowa Wild Turkeys starting in the U3 region of the LTR (Long Terminal Repeat) and extending into part of the Matrix.


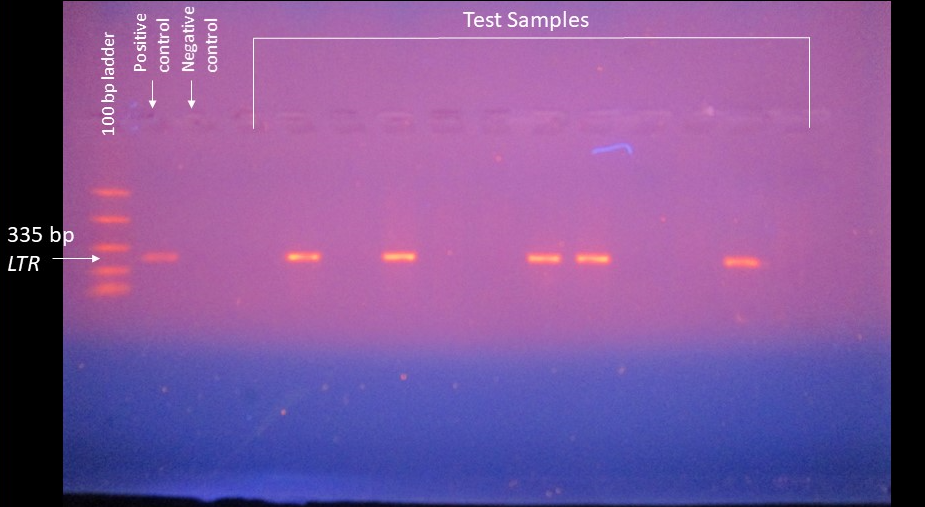

Supplement: S1 File — (DOCX) [file pone.0296856.s002.docx]
